# Long-term exposure to a mixture of industrial SO_2_, NO_2_, and PM_2.5_ and anti-citrullinated protein antibody positivity

**DOI:** 10.1186/s12940-020-00637-3

**Published:** 2020-07-29

**Authors:** Naizhuo Zhao, Audrey Smargiassi, Marianne Hatzopoulou, Ines Colmegna, Marie Hudson, Marvin J. Fritzler, Philip Awadalla, Sasha Bernatsky

**Affiliations:** 1 Division of Clinical Epidemiology, McGill University Health Centre, Montreal, QC Canada; 2 Département de Santé Environnementale et de Santé au Travail, Université de Montréal, Montréal, QC Canada; 3 Institut National de Santé Publique du Québec, Montréal, QC Canada; 4 Centre de Recherche en Santé Publique de l’Université de Montréal (CReSP), Montréal, QC Canada; 5 Department of Civil Engineering, University of Toronto, Toronto, ON Canada; 6 Department of Medicine, McGill University, Montréal, QC Canada; 7 Division of Rheumatology, McGill University Health Center, Montréal, QC Canada; 8 Lady Davis Institute for Medical Research, Jewish General Hospital, Montréal, QC Canada; 9 Department of Medicine, Cumming School of Medicine, University of Calgary, Calgary, AB Canada; 10 Ontario Institute for Cancer Research, Toronto, ON Canada; 11 Department of Molecular Genetics, University of Toronto, Toronto, ON Canada; 12 Centre for Outcomes Research & Evaluation, 5252 boul de Maisonneuve Ouest, (3F.51), Montreal, QC H4A 3S5 Canada

**Keywords:** Anti-citrullinated protein antibodies (ACPA), Industrial air pollutants, Regional fine particles matter (PM_2.5_), Weighted quantile sum (WQS) regression, California puff (CALPUFF) model

## Abstract

**Background:**

Studies of associations between industrial air emissions and rheumatic diseases, or diseases-related serological biomarkers, are few. Moreover, previous evaluations typically studied individual (not mixed) emissions. We investigated associations between individual and combined exposures to industrial sulfur dioxide (SO_2_), nitrogen dioxide (NO_2_), and fine particles matter (PM_2.5_) on anti-citrullinated protein antibodies (ACPA), a characteristic biomarker for rheumatoid arthritis (RA).

**Methods:**

Serum ACPA was determined for 7600 randomly selected CARTaGENE general population subjects in Quebec, Canada. Industrial SO_2_, NO_2_, and PM_2.5_ concentrations, estimated by the California Puff (CALPUFF) atmospheric dispersion model, were assigned based on residential postal codes at the time of sera collection. Single-exposure logistic regressions were performed for ACPA positivity defined by 20 U/ml, 40 U/ml, and 60 U/ml thresholds, adjusting for age, sex, French Canadian origin, smoking, and family income. Associations between regional overall PM_2.5_ exposure and ACPA positivity were also investigated. The associations between the combined three industrial exposures and the ACPA positivity were assessed by weighted quantile sum (WQS) regressions.

**Results:**

Significant associations between individual industrial exposures and ACPA positivity defined by the 20 U/ml threshold were seen with single-exposure logistic regression models, for industrial emissions of PM_2.5_ (odds ratio, OR = 1.19, 95% confidence intervals, CI: 1.04–1.36) and SO_2_ (OR = 1.03, 95% CI: 1.00–1.06), without clear associations for NO_2_ (OR = 1.01, 95% CI: 0.86–1.17). Similar findings were seen for the 40 U/ml threshold, although at 60 U/ml, the results were very imprecise. The WQS model demonstrated a positive relationship between combined industrial exposures and ACPA positivity (OR = 1.36, 95% CI: 1.10–1.69 at 20 U/ml) and suggested that industrial PM_2.5_ may have a closer association with ACPA positivity than the other exposures. Again, similar findings were seen with the 40 U/ml threshold, though 60 U/ml results were imprecise. No clear association between ACPA and regional overall PM_2.5_ exposure was seen.

**Conclusions:**

We noted positive associations between ACPA and industrial emissions of PM_2.5_ and SO_2_. Industrial PM_2.5_ exposure may play a particularly important role in this regard.

## Introduction

Air pollution is a major risk factor for cardiorespiratory and chronic airway diseases [1–3]. By contrast, studies of air pollution and rheumatic diseases and/or their serologic biomarkers are relatively few, and conclusions from these limited studies are inconsistent [4]. Laboratory studies have shown that ambient air pollutants inhaled and deposited in the lungs can increase airway inflammation [5, 6], triggering systemic autoimmune responses (and possibly facilitating the development of autoimmune rheumatic disease) [7]. However, positive associations between air pollution exposure and autoimmune responses and/or rheumatic disease onset have not always been observed in observational studies [8].

Rheumatoid arthritis (RA) is the most common world-wide chronic inflammatory disease and causes great disability [9]. Anti-citrullinated protein antibodies (ACPA) are a characteristic finding in RA, often predating clinical manifestations of the disease by years [10]. We previously reported that exposure to industrial air emissions, e.g. sulfur dioxide (SO_2_) and fine particles matter (PM_2.5_), was associated with increased probability of ACPA positivity in a general population sample [11]. However, in that study a rough proxy of exposure (i.e., distance to major industrial emitters) was used and the number of positive ACPA cases was relatively small.

As well, people are exposed to mixtures of multiple pollutants, yet the joint effects of different air pollutants have not been previously considered in studies of air pollution and rheumatic autoimmune diseases and/or serologic biomarkers. Concentrations of regional ambient air pollutants, and especially industrial air pollutants, are usually correlated in space [12], since these pollutants are often derived from the same sources (e.g. road traffic and factories). Hence, special analytic approaches that can effectively address collinearity should be used for exploring the associations between inter-correlated exposures and the outcome of interest [13].

Given the paucity of studies on individual air pollutant exposures and rheumatic diseases, and the absence of prior evaluations of rheumatic-related antibodies and multi-pollutant mixtures, we expanded our previous analyses within a population-based cohort in Quebec, Canada [11], to investigate associations between exposures to three industrial air pollutants (i.e. SO_2_, nitrogen dioxide - NO_2_, and PM_2.5_) and ACPA positivity. In this new effort, we doubled the sample size, used more accurate pollutant estimates derived from a three-dimensional atmospheric model (California Puff, CALPUFF), and evaluated multiple thresholds for defining ACPA positivity. Moreover, a weighted quantile sum (WQS) regression model [14] was used to detect the joint effect of the multi-pollutant exposures on ACPA positivity.

## Methods

### Study population and sera samples

Our analyses were based on the CARTaGENE cohort (www.cartagene.qc.ca), which is composed of 43,000 general population subjects aged between 40 to 69 years old, with residential history equal to or longer than 5 years in Quebec, Canada. CARTaGENE is part of the Canadian Partnership for Tomorrow Project, a prospective cohort study created as a population-health research platform for assessing the effect of genetics, behaviour, family health history and environment (among other factors) on chronic diseases [15]. Participants in the CARTaGENE cohort were randomly selected from the provincial health insurance database and invited to participate. At baseline, CARTaGENE data were generated at enrolment and included a wide range of health-related variables such as demographics, medical history, lifestyle factors like smoking, and self-reported RA (past diagnosed by physicians) [16], and baseline serum samples were biobanked. The original smoking variable in the CARTaGENE baseline dataset had four categories that were daily, past, occasional, and never smoking. We incorporated the past smoking category into the occasional smoking category because only 4.0% of subjects were past smokers. Thus, in our analyses, individuals reporting anything other than daily or never smoking were categorized as occasional/past smokers.

For the current study, we selected a random sample of 7600 individuals from the first CARTaGENE recruitment wave (enrolled over 2009–2010). This sample size is twice as large as that of our previous study [11]. Biobanked serum samples were assessed for ACPA positivity by chemiluminescence immunoassay (CCP3.0; Inova Diagnostics, San Diego, CA, USA) at the Mitogen Advanced Diagnostics Lab in Calgary, Alberta. ACPA positivity was defined initially on the basis of test results ≥20 U/ml [17]. In sensitivity analyses, two other thresholds were also used, to classify all positive ACPA outcomes as weak (20–39 U/ml), moderate (40–59 U/ml), and strong (≥60 U/ml) positive titres [18].

### Air pollution exposures

CALPUFF is an advanced dispersion modeling system, which can simulate the effects of spatiotemporally varying meteorological conditions on transport, transformation, and dissipation of air pollutants [19]. The modeling system consists of three major components namely CALMET (a three-dimensional meteorological model), CALPUFF (an air quality dispersion model), and CALPOST (a post-processing package). The CALPUFF system is recommended by the United States Environmental Protection Agency to assess long-range tracking of air pollutants and has been extensively used to map regional SO_2_, NO_2_, and particulate matter concentrations in Canada, the United States, and other countries [20–25]. In this study, using industrial emissions reported to the National Pollutant Release Inventory [26], industrial SO_2_, NO_2_, and PM_2.5_ annual average levels for 2005–2010 were modeled by the CALPUFF at the locations of Quebec’s six-digit postal codes and then were assigned to each subject based on his/her postal code at the time of CARTaGENE enrollment (when blood samples were taken). Please see the paper of Buteau et al. (2020) [27] for details of using the CALPUFF modelling system to estimate industrial SO_2_, NO_2_, and PM_2.5_ annual average concentrations.

The annual average regional (overall but not only industrial) PM_2.5_ concentration estimates were retrieved from the Atmospheric Composition Analysis Group at Dalhousie University. The PM_2.5_ concentrations were first estimated at the 10 km resolution using the GEOS (Goddard Earth Observing System) chemical-transport-model and the satellite-derived aerosol optical depth data [28]. These coarse gridded PM_2.5_ products were further resampled to the 1 km spatial resolution by a geographical weighted regression model and additional covariates, e.g. elevation, vegetation index, and distance to urban areas [29]. Similar to the industrial SO_2_, NO_2_, and PM_2.5_ exposures, the average regional (all-sector) PM_2.5_ estimates for 2005–2010 extracted from the above raster dataset were assigned to all the participants based on their six-digit postal codes at the time of the cohort enrollment. Since all participants entered into the CARTaGENE cohort during 2009 to 2010 and had residential history in Quebec equal to or longer than 5 years, we were assured that the participants have been in Quebec from at least 2005. Thus, as in our previous study [30], we selected the exposure time window of 2005–2010 to ensure that subjects’ assigned long-term air pollution exposures represented their actual exposures.

### Standard logistic regression models

We first used three separate single-exposure standard logistic regression models, adjusting for age, sex, ancestry, smoking, and family income (see Table 1 for the detailed categories of the covariates), to detect the associations between individual industrial SO_2_, NO_2_, and PM_2.5_ exposures and ACPA positivity (defined by the 20 U/ml threshold). These covariates were chosen as they may be potential effect modifiers (e.g. sex) or confounders (e.g. age, French Canadian ancestry, family income, and smoking) of relationships between variations in air pollution and serologic positivity [11]. The single-exposure logistic regression adjusting for the same covariates was also conducted for regional overall PM_2.5_ exposure, to examine whether the same air pollutant from different (i.e. regional overall vs. industrial) emission sources would produce different effects on ACPA positivity. Next, we increased the threshold of defining ACPA positivity to 40 U/ml and 60 U/ml, and used the above single-exposure logistic regression models in sensitivity analyses. We did not use multi-exposure logistic regressions to investigate the associations of combined exposures to industrial SO_2_, NO_2_, and PM_2.5_, because concentrations of the three industrial air pollutants are closely correlated in space (see the Results section for specific correlation coefficients). To see whether air pollution exposures have different effects on ACPA and RA, we also used the standard logistic regression models, adjusting for age, sex, ancestry, smoking, and family income, to detect the associations between RA and individual industrial SO_2_, NO_2_, and PM_2.5_ and regional overall PM_2.5_ exposures.

**Table 1:** Baseline characteristics of the subjects and distributions of pollutants according to antibody outcomes

ACPA outcome		Positive			Negative (< 20 units/ml)
		Strong (≥60 units/ml)	Moderate (40–59 units/ml)	Weak (20–39 units/ml)	
**Number of subjects (%)**		134 (1.8)	158 (2.1)	494 (6.5)	6788 (89.6)
**Mean age**^**a**^**(standard deviation)**		55.1 (7.5)	53.8 (7.8)	54.6 (7.6)	54.0 (7.7)
**Female, N (%)**		76 (56.7)	88 (55.7)	247 (50.0)	3441 (50.7)
**Ancestry, N (%)**	**French Canadian**	96 (71.6)	103 (65.2)	330 (66.8)	4571 (67.3)
	**Other**	38 (28.4)	55 (34.8)	164 (33.2)	2217 (32.7)
**Smokers**^**b**^**,N (%)**	**Never**	45 (33.6)	68 (43.0)	225 (45.5)	2710 (39.9)
	**Occasional**	69 (51.5)	67 (42.4)	208 (42.1)	3141 (46.2)
	**Daily**	20 (14.9)	23 (14.6)	59 (11.9)	913 (13.5)
**Annual income level, N (%)** **(Canadian $)**	**< 25,000**	13 (9.7)	19 (12.0)	44 (8.9)	631 (9.3)
	**25,000 to 49,999**	29 (21.6)	34 (21.5)	94 (19.0)	1363 (20.1)
	**50,000 to 74,999**	32 (23.9)	29 (18.4)	111 (22.5)	1447 (21.3)
	**75,000 to 149,999**	38 (28.4)	49 (31.0)	151 (30.6)	2233 (32.9)
	**> 150,000**	15 (11.1)	15 (9.5)	64 (13.0)	782 (11.5)
**Range, mean, and standard deviation of exposure variables**	**Industrial SO**_**2**_**(ppb**)	0.64–19.57, 2.89, 2.34	0.62–71.19, 2.91, 5.71	0.61–17.14, 2.69, 1.94	0.34–60.98, 2.56, 2.15
	**Industrial NO**_**2**_**(ppb**)	0.16–3.01, 1.14, 0.56	0.27–6.05, 1.25, 0.73	0.26–4.06, 1.13, 0.57	0.12–7.76, 1.16, 0.52
	**Industrial PM**_**2.5**_**(**μg**/m**^**3**^**)**	0.06–2.87, 0.27, 0.39	0.05–14.09, 0.28, 1.14	0.05–3.12, 0.23, 0.32	0.03–11.17, 0.21, 0.36
	**Overall PM**_**2.5**_**(μg/m**^**3**^**)**	5.27–14.85, 11.24, 3.06	5.58–14.85, 11.55, 2.97	5.22–14.85, 11.44, 3.02	5.13–14.85, 11.76, 2.90

^a^ Age is a continuous numeric variable in the standard logistic and Weighted Quantile Sum (WQS) regression models

^b^ Missing data existed for the covariates smoking and income, and thus the summed number of daily, occasional, and never smokers is slightly smaller than the total number of population subjects involved in the analysis

### The WQS regression models

The joint association of the three highly correlated industrial air pollutants with ACPA positivity was explored by the WQS regression method and quantitatively assessed by a WQS index [14]. The WQS approach supposes that all the studied exposures have the same direction (positive or negative) effects on the disease outcome. Magnitudes of the individual effects of different exposures are quantified by a set of weights. Each of the weights is constrained to be between 0 and 1, and all of the weights are summed to 1. The weights were multiplied by the scored quartiles of the individual exposures, and then were accumulated to obtain the WQS index.

To calculate the weights, we first performed natural logarithm transformations on the three exposure variables to ensure each had similar scales. Then, we divided the sample into a training and a validation datasets using a split proportion of 4:6. This proportion was adopted by the previous WQS studies [14, 31] because leaving more subjects in the validation dataset tends to increase robustness for calculating the significance of the WQS index [14]. A total of *B* = 100 bootstrap samples were generated from the training dataset to estimate the unknown weight *w*_*i*_ (*i* denoting one of the industrial air pollutants) by maximizing the likelihood of the weighted index function:

1 $$ {\left.g\left(\mu \right)={\beta}_0+{\beta}_1\left({\sum}_{i=1}^3{w}_i{q}_i\right)+{\beta}^Tz\right|}_b\kern0.5em \left(b=1,2,\dots, 100\right) $$

where *g*(·) is a logit link function for the binary outcome of a positive (or negative) ACPA, *z* denotes a vector of potential confounders or effect modifiers (i.e. age, sex, French Canadian ancestry, smoking, and family income), *β* is the coefficient vector of the covariates, *β*_*0*_ is the intercept, *q* represents a quartile of the logarithmically transformed exposure. The term ${\sum}_{i=1}^3{w}_i{q}_i$ represents the weighted index and *β*_*1*_ is its regression coefficient. Let $WQS={\sum}_{i=1}^3{w}_i{q}_i$, and thus the eq. 1 can be simplified as.

2 $$ g\left(\mu \right)={\beta}_0+{\beta}_1 WQS+{\beta}^Tz $$

The odds ratio (OR) associated with a quartile increase in all of the three logarithmically transformed exposures (i.e. the WQS index) is equal to exponentiated *β*_*1*_.

The specific WQS regression was implemented using the “gWQS” package [32] in the R statistical computing environment. Similar to the single-exposure logistic regressions, the WQS regressions were conducted three times for positive ACPA outcomes defined by the three thresholds (i.e. 20 U/ml or higher, 40 U/ml or higher, and 60 U/ml or higher).

RA affects less than 1% of the general population of Quebec [33]. After splitting our sample into a training and a validation datasets, we did not have enough RA cases in either dataset for a reliable fitting or validation. Thus, we did not use WQS regression to detect the relationship between combined industrial exposures and RA in this study.

## Results

In the total 7600 subjects the mean age at cohort entry was 54.1 years (standard deviation, SD =7.7 years) and 3859 (50.8%) were female. Approximately two-third (67.3%) of the subjects were French Canadians. Over 40 % (*N* = 3053, 40.2%) of the subjects were never smokers, 1020 (13.4%) were daily smokers, 3492 (45.9%) were occasional/past smokers, and the remainder (*N* = 26) had missing smoking data. Only 9.3% of the population subjects lived below the lowest household income level (i.e. < 25,000 Canadian dollars per year) while 11.5% belonged to the highest level for income (i.e. ≥150,000 Canadian dollars per year). Detailed comparisons among the strong, moderate, and weak ACPA positive and negative subjects are presented in Table 1. A total of 201 subjects in our sample reported physician-diagnosed RA when they entered the cohort, and 37 individuals had both RA and positive ACPA. Furthermore, 24 of the 37 individuals had ACPA ≥60 U/ml.

The interquartile ranges of the logarithmically transformed industrial SO_2_, NO_2_, and PM_2.5_ exposures were 1.34 ppb, 1.04 ppb, and 1.58 μg/m^3^, respectively. Pearson’s correlations coefficients (*r*) indicated that besides a moderate correlation between industrial PM_2.5_ and regional overall PM_2.5_ concentrations (*r* = − 0.13, *p* < 0.001, 95% confidence intervals, CI -0.16 – − 0.11), industrial PM_2.5_ levels were strongly correlated to those of industrial SO_2_ (*r* = 0.96, *p* < 0.001, 95% CI: 0.96–0.97) and moderately to NO_2_ (*r* = 0.19, *p* < 0.001, 95% CI: 0.17–0.21); the concentration of SO_2_ was also moderately correlated with NO_2_ (*r* = 0.35, *p* < 0.001, 95% CI: 0.33–0.37).

As presented in Table 2, clearly positive associations between industrial SO_2_ (OR: 1.03, 95% CI: 1.00–1.06) and PM_2.5_ (OR: 1.19, 95% CI: 1.04–1.36) exposures and ACPA positivity were observed from the standard single-exposure regression analyses, when the ACPA positivity was defined by the 20 U/ml threshold. With the threshold increased to 40 U/ml, the positive associations of industrial SO_2_ (OR: 1.03, 95% CI: 1.00–1.07) and PM_2.5_ (OR: 1.21, 95% CI: 1.02–1.42) exposures with ACPA positivity were similar. However, when the ACPA threshold was further increased to 60 U/ml, the point estimates were similar but the 95% CIs became wider due to very low numbers of cases (industrial SO_2_ OR: 1.03, 95% CI: 0.98–1.08 and industrial PM_2.5_ OR: 1.17, 95% CIs: 0.92–1.48). Industrial NO_2_ and regional overall PM_2.5_ exposures were not clearly associated with ACPA positivity, regardless of the thresholds used to define positivity (Table 2). Positive ACPA was more common in subjects of older age (as is expected, given that both RA and ACPA are more common in older individuals) [34]. Due to low power, we did not see a clear relationship between RA and any air pollutant exposure (see Table S1). A few previous studies (e.g. [35–38]) have found that smoking increased the risk of developing ACPA-positive RA, but we did not find a clear relationship of smoking with either ACPA positivity or RA (Table S2).

**Table 2:** Adjusted OR (95% CIs) from the single-pollutant logistic regression models for ACPA positivity

Exposure variable	Positivity: ≥60 units/ml (N positive = 134)	Positivity: ≥40 units/ml (N positive = 292)	Positivity: ≥20 units/ml (N positive = 786)
**Industrial SO**_**2**_	1.03 (0.98–1.08)	1.03 (1.00–1.07)*	1.03 (1.00–1.06)*
**Industrial NO**_**2**_	0.90 (0.63–1.28)	1.14 (0.91–1.41)*	1.01 (0.86–1.17)*
**Industrial PM**_**2.5**_	1.17 (0.92–1.48)	1.21 (1.02–1.42)	1.19 (1.04–1.36)
**Overall PM**_**2.5**_	0.94 (0.89–1.01)	0.95 (0.91–1.01)	0.98 (0.95–1.01)

Adjusted ORs (95% CI) for industrial SO_2_ and NO_2_ are per 1 ppb increase while they are reported per 1 μg/m^3^ increase for regional and overall PM_2.5_ levels. Variables adjusted for include age, sex, ancestry, smoking, and annual income level. *Statistically significant associations include industrial PM_2.5_ (where 95% CIs exclude the null value) and industrial SO_2_ (where 95% CIs just barely include the null value)

The WQS index (i.e. the mixture of the three industrial air emissions) was significantly correlated with ACPA positivity defined by the 20 U/ml threshold. Specifically, an interquartile increase in the WQS index led to an increase of 1.36 (95% CI: 1.10–1.69) in the odds of ACPA positivity. With the positivity threshold increased to 40 U/ml, the positive association between the combined logarithmically transformed exposure of the three industrial air pollutants and ACPA positivity was still apparent (OR = 1.43, 95% CI: 1.05–1.96). When the ACPA positivity was defined by a higher threshold of 60 U/ml, due to low numbers of cases, the association between the WQS index and ACPA positivity became less clear (OR = 1.33, 95% CI: 0.85–2.10). Regardless of the threshold for ACPA positivity, industrial PM_2.5_ was always the most heavily weighted while the industrial NO_2_ was the most lightly weighted in the index (Table 3).

**Table 3:** Adjusted OR (95% CIs) from the weighted quantile sum (WQS) regressions for ACPA positivity

Threshold of ACPA positivity	OR (95% CI)	Weight		
		SO_**2**_	NO_**2**_	PM_**2.5**_
**20 units/ml (N positive = 786)**	1.36 (1.10–1.69)	0.12	0.00	0.88
**40 units/ml (N positive = 292)**	1.43 (1.05–1.96)	0.28	0.18	0.54
**60 units/ml (N positive = 134)**	1.33 (0.85–2.10)	0.22	0.14	0.64

Adjusted ORs are per increase of an interquartile range of the logarithmically transformed industrial air pollutants. Variables adjusted for include age, sex, ancestry, smoking, and annual income level

## Discussion

Exposure to ambient air pollutants may induce pulmonary oxidative stress and inflammation [39, 40] and consequently trigger autoimmune responses which could favor the development of RA and related diseases [7, 41, 42]. However, results of early epidemiologic studies have not always supported this hypothesis. Although, Hart et al. (2013) [43] found that exposure to NO_2_ from road traffic is likely to increase risk of RA incidence using a Swedish general population cohort, positive associations between RA incidence and exposure to NO_2_ and PM_2.5_ were not observed by De Roos et al. (2014) [8] in British Columbia, Canada. Gan et al. (2013) [9] also did not find conclusive associations between ambient particulate matter exposure and RA-related antibodies in first-degree relatives of RA patients in the United States, but a clear association between ambient NO_2_ and RA was found by Chang et al. (2016) [44] in Taiwan. Due in part to low power, we did not see a clear relationship between RA and any air pollutant exposure. Linking baseline CARTaGENE data with administrative data could be a way to generate follow-up data, which may allow us to obtain information on new cases of RA within CARTaGENE, to better study the relationships between RA and air pollution exposures in the future [33].

Several previous studies using cohorts from Europe reported that smoking could increase the risk of ACPA-positive RA while a conclusive association between smoking and ACPA-negative RA was not observed [36–38]. In our study, only 4.7% of ACPA-positive subjects reported a physician diagnosis of RA before they entered the cohort. Thus, failure to find associations between smoking and ACPA positivity dose not necessarily contradict the findings from Europe [35–38] and is consistent with our previous finding in Quebec [11].

In our mixed-pollutant analyses, regardless of the threshold for ACPA positivity, industrial PM_2.5_ appeared to be the most influential exposure, while exposure to industrial NO_2_ was the least influential. In an earlier study using CARTaGENE data, we found that exposures to industrial SO_2_ and PM_2.5_ were associated with ACPA positivity, but no clear associations were seen with industrial NO_2_ and ambient PM_2.5_ [11]_._ In the current study, we reinforced these findings with twice the sample size, and more accurate estimates of exposure to industrial air emission (since the prior study used simple distance to major industrial emitters). Most importantly, the use of the WQS model allowed us to assess the combined association of all three industrial emissions and to quantify different contributions of the individual emissions on ACPA positivity, which is more representative of how people are always exposed to multiple, correlated air pollutants. We found similar associations between ACPA and industrial PM_2.5_ and SO_2_, at both low and medium titres, although limiting positivity to very higher titres led to imprecise results.

Surface chemistry of industrial ambient particulate matter is likely to be more toxic than that of regional overall ambient particulate matter [45], which may explain why ACPA positivity was associated with industrial PM_2.5_ exposure but not ambient PM_2.5_ exposure. Additionally, we found that the industrial PM_2.5_ concentration was negatively correlated with the regional overall PM_2.5_ concentration. In other words, individuals exposed to higher ambient PM_2.5_ levels are less likely to be exposed to high industrial PM_2.5_ concentrations. This might be explained by the fact that industrial emitters of PM_2.5_ tend to be located away from high-traffic areas (since motor vehicles account for the majority of ambient PM_2.5_ levels to which people are exposed) [46].

When studying the joint effects of multiple air pollutant exposures it may be preferable to use the WQS approach rather than standard logistic regression models, in order to avoid the problem of collinearity. However, the WQS method has a critical restriction, in that if the studied exposures have effects on the disease outcome that differ in direction (i.e. positive versus negative), the model will not converge. The Bayesian kernel machine regression (BKMR) [47] is an alternative method to study the combined effects of multiple correlated exposures on binary disease outcomes, without this critical restriction. However, the computing time of fitting a BKMR model increases exponentially with an increase in the number of subjects. By contrast, the WQS model is much more efficient for a large sample, which prompted us to choose the WQS and not the BKMR approach in this study.

Participants in the CARTaGENE cohort were all aged between 40 to 69 years old. That is a potential limitation of this study, since younger individuals may be more susceptible to the adverse health effects from air pollution [42]. However, people in the 40 to 69 age group are less mobile than younger (e.g. college age) ones. This may be beneficial in terms of reducing errors when postal codes at a single point in time are used to assign exposure information like in this study (where highly mobile populations may be subject to more misclassification of exposure).

In this study, the average level of industrial NO_2_ in the population under study was very low, and variation in the industrial NO_2_ concentration across Quebec is small, which may be a reason why we failed to observe a clear association between industrial NO_2_ exposure and ACPA positivity. Thus, additional studies including younger populations and conducted in higher industrial NO_2_ regions may help reinforce or refute the current findings. Besides air pollution exposures, occupational dust exposures (e.g. asbestos, silica, and carbon nanoparticles) are also likely to be associated with ACPA positivity and RA [48, 49]. Thus, occupation may need to be added as a covariate in the next studies regarding industrial air pollution exposures and ACPA. In addition, further study of air pollution and RA onset may be informative, particularly if more sophisticated approaches (such as WQS regression or BKMR) are employed. Another future direction may be to examine RA-related manifestations, such as pulmonary disease, and air pollution [50].

## Conclusions

Using a larger sample, more accurate exposure estimates, and more detailed positivity definition than in the past, this study reinforced our previous findings that exposures to industrial sources of SO_2_ and PM_2.5_ tend to increase the probability of ACPA positivity. No clear association between ACPA positivity and industrial NO_2_ or regional overall PM_2.5_ exposure was detected. The use of the WQS approach allowed us to produce new findings concerning positive associations between mixtures of the industrial air pollutants, and suggested that the effects of industrial PM_2.5_ exposure on ACPA positivity may be more important than that of industrial SO_2_ exposure.

## Supplementary information

**Additional file 1: Table S1.** Adjusted OR (95% CIs) from the single-pollutant logistic regression models for RA. **Table S2.** Adjusted OR (95% CIs) from the single-pollutant logistic regression models for ACPA positivity defined by the 20 unit/ml threshold.

## Data Availability

The datasets used in the current study are available from the corresponding author on reasonable request.
