# Supplementary material for: Long-term exposure to a mixture of industrial SO2, NO2, and PM2.5 and anti-citrullinated protein antibody positivity
Source: Environ Health. 2020 Jul 29;19:86. doi: 10.1186/s12940-020-00637-3 (PMC7391811; doi:10.1186/s12940-020-00637-3)
Supplement: Supplementary file 1 — Additional file 1: Table S1. Adjusted OR (95% CIs) from the single-pollutant logistic regression models for RA. Table S2. Adjusted OR (95% CIs) from the single-pollutant logistic regression models for ACPA positivity defined by the 20 unit/ml threshold. [file 12940_2020_637_MOESM1_ESM.docx]

**Supplementary material**

**Long-term exposure to a mixture of industrial SO_2_, NO_2_, and PM_2.5_ and anti-citrullinated protein antibody positivity**

Naizhuo Zhao^1^, Audrey Smargiassi^2,3,4^, Marianne Hatzopoulou^5^, Ines Colmegna^6,7^, Marie Hudson^6,8^, Marvin J. Fritzler^9^, Philip Awadalla^10,11^, Sasha Bernatsky^1,6,7*^

1. Division of Clinical Epidemiology, McGill University Health Centre, Montreal, QC, Canada.

2. Département de Santé Environnementale et de Santé au Travail, Université de Montréal, Montréal, QC, Canada.

3. Institut National de Santé Publique du Québec, Montréal, QC, Canada.

4. Centre de Recherche en Santé Publique de l’Université de Montréal (CReSP), Montréal, QC, Canada.

5. Department of Civil Engineering, University of Toronto, Toronto, ON, Canada.

6. Department of Medicine, McGill University, Montréal, QC, Canada.

7. Division of Rheumatology, McGill University Health Center, Montréal, QC, Canada.

8. Lady Davis Institute for Medical Research, Jewish General Hospital, Montréal, QC, Canada.

9. Department of Medicine, Cumming School of Medicine, University of Calgary, Calgary, AB, Canada.

10. Ontario Institute for Cancer Research, Toronto, ON, Canada.

11. Department of Molecular Genetics, University of Toronto, Toronto, ON, Canada.

*Corresponding author. Centre for Outcomes Research & Evaluation, 5252 boul. de Maisonneuve Ouest, (3F.51) Montreal, Quebec, H4A 3S5, Canada. Tel: 514 934-1934 ext. 44710, E-mail address: [sasha.bernatsky@mcgill.ca](mailto:sasha.bernatsky@mcgill.ca) (S. Bernatsky).

**Table S1. Adjusted OR (95% CIs) from the single-pollutant logistic regression models for RA.**

| **Exposure variable** | **Industrial SO_2_** | **Industrial NO_2_** | **Industrial PM_2.5_** | **Overall PM_2.5_** |
| --- | --- | --- | --- | --- |
| **OR**  **(95% CIs)** | 0.93  (0.82 – 1.04) | 1.11  (0.85 – 1.43) | 0.44  (0.14 – 1.37) | 1.01  (0.96 – 1.08) |

Adjusted ORs (95% CI) for industrial SO_2_ and NO_2_ are per 1 ppb increase while they are reported per 1 µg/m^3^ increase for regional and overall PM_2.5_ levels. Variables of adjustment are age, sex, ancestry, smoking, and annual income level.

**Table S2. Adjusted OR (95% CIs) from the single-pollutant logistic regression models for ACPA positivity defined by the 20 unit/ml threshold.**

| **Covariates** | | **Industrial SO_2_** | **Industrial NO_2_** | **Industrial PM_2.5_** | **Regional overall PM_2.5_** |
| --- | --- | --- | --- | --- | --- |
| **Exposure variable** | | 1.03  (1.00 – 1.06) | 1.01  (0.86 – 1.17) | 1.19  (1.04 – 1.36) | 0.98  (0.95 – 1.01) |
| **Age** | | 1.01  (1.00 – 1.02) | 1.01  (1.00 – 1.02) | 1.02  (1.00– 1.03) | 1.01  (1.00– 1.03) |
| **Sex** | **Male** | Reference | | | |
|  | **Female** | 1.03  (0.88– 1.21) | 1.03  (0.88 – 1.21) | 1.03  (0.88 – 1.21) | 1.04  (0.86 – 1.24) |
| **Ancestry** | **French Canadian** | Reference | | | |
|  | **Other** | 0.99  (0.83 – 1.17) | 0.98  (0.82 – 1.16) | 0.99  (0.83 – 1.18) | 1.04  (0.85 – 1.27) |
| **Smoking** | **Never** | Reference | | | |
|  | **Occasional**  **/past** | 0.98  (0.76 – 1.26) | 0.98  (0.76 – 1.25) | 0.98  (0.76 – 1.26) | 1.01  (0.76 – 1.34) |
|  | **Daily** | 1.11  (0.86 – 1.42) | 1.10  (0.86 – 1.42) | 1.10  (0.86 – 1.42) | 1.17  (0.88 – 1.56) |
| **Annual income level**  **(Canadian $)** | **<25,000** | Reference | | | |
|  | **25,000 to 49,999** | 0.98  (0.72 – 1.33) | 0.98  (0.73 – 1.32) | 0.98  (0.72 – 1.32) | 0.92  (0.66 – 1.29) |
|  | **50,000 to 74,999** | 1.02  (0.76 – 1.37) | 1.02  (0.76 – 1.37) | 1.02  (0.76 – 1.37) | 0.96  (0.68 – 1.35) |
|  | **75,000 to 149,999** | 0.94  (0.71 – 1.26) | 0.94  (0.70 – 1.25) | 0.94  (0.71 – 1.26) | 0.90  (0.65 – 1.24) |
|  | **>150,000** | 1.06  (0.75 – 1.48) | 1.05  (0.75 – 1.47) | 1.06  (0.75 – 1.48) | 0.89  (0.60 – 1.33) |

Adjusted ORs (and 95% CIs) for industrial SO_2_ and NO_2_ are per 1 ppb increase while they are reported per 1 μg/m^3^ increase for industrial or regional overall PM_2.5_.
